# COVID-19 Pandemic-Related Restrictions: Factors That May Affect Perinatal Maternal Mental Health and Implications for Infant Development

**DOI:** 10.3389/fped.2022.846627

**Published:** 2022-05-12

**Authors:** Theano Kokkinaki, Eleftheria Hatzidaki

**Affiliations:** ^1^Child Development and Education Unit, Laboratory of Applied Psychology, Department of Psychology, University of Crete, Rethymno, Greece; ^2^Department of Neonatology, Neonatal Intensive Care Unit (NICU), School of Medicine, University of Crete, Rethymno, Greece

**Keywords:** COVID-19 pandemic, maternal mental health, neonate/infant development, family functioning, maternal health care policy, birth experience, NICU, breastfeeding

## Abstract

This review aims to discuss the factors that may affect maternal mental health and infant development in COVID-19 pandemic condition. Toward this direction, the two objectives of this review are the following: (a) to discuss possible factors that may have affected negatively perinatal mental health through the pandemic-related restrictions; and (b) to present the implications of adversely affected maternal emotional wellbeing on infant development. We conclude that the pandemic may has affected maternal mental health with possible detrimental effects for the infants of the COVID-19 generation. We highlight the need for evidence-based interventions to be integrated within the health system for prenatal and postpartum care in an effort to promote maternal mental health and infant development.

## Introduction

COVID-19 pandemic constitutes a major threat to global human health and a worldwide traumatic experience (1, 2). During the 2 years of the COVID-19 pandemic, million infants were born to mothers and families who have experienced tremendous stress and change in their daily lives and environments due to the pandemic (3–5)].

According to the World Health Organization (WHO), maternal mental health is defined as “a state of wellbeing in which a mother realizes her own abilities, can cope with the normal stresses of life, can work productively and fruitfully, and is able to make a contribution to her community” [as cited in Engle (6)]. Perinatal mental disorders constitute the commonest complications of child-bearing, and they are associated with high levels of maternal and fetal/infant morbidity and mortality (7). For women in the perinatal period, research has identified two major-pandemic–related stress domains: stress associated with feeling unprepared for birth and stress related to fears of perinatal infection (8). What is more, in the first months of COVID-19 pandemic, evidence from around the world^1^ indicated that pregnant women suffer from high prevalence of anxiety (ranging from 21.7 to 78.4%), depression (ranging from 17 to 56.3%) and post-traumatic stress disorder (9–13). During COVID-19, the overall prevalence of anxiety and depression among pregnant women was 40 and 27%, respectively. Though the levels of anxiety and^2^ depression during pregnancy vary across different countries, in general prenatal depression is estimated to affect 9–18% of pregnant women at any given time during pregnancy (14, 15) while 5–13% of pregnant women suffer from anxiety symptoms (10, 11). Thus, evidence suggests that symptoms of maternal mental disorders have become more common during the pandemic (9–13). In the meanwhile, prenatal psychopathological diagnoses have been rarely investigated (13) and only a limited number of studies analyzed longitudinally anxiety and depression symptoms of pregnant women in the course of the lockdown. Despite contrasting results, findings show that ongoing COVID-19 pandemic may aggravate anxiety and depression symptoms of pregnant women (9, 12, 16).

COVID-19 pandemic coincides with sensitive time windows of heightened plasticity, such as pregnancy and neonatal life (1, 2). The significance of adversely affected perinatal maternal mental health as a potential risk factor for infant development has been emphasized (17). Prenatal stress affects the development of fetal systems (14, 15, 18–21). These fetal systems are also potentially related factors and causes of neuropsychiatric disorders (depression, anxiety, behavioral dysfunction, attention-deficit hyperactivity disorder, autism spectrum disorder) in children (21). Further, symptoms of maternal mental disorders have been linked with delays and poor infant motor, social, cognitive, and language development and difficulties in emotional self-regulation [see (22, 23) for reviews].

In addition, face mask wearing by caregivers in daily interactions with their infants may affect negatively: (a) infants' abilities related to social and emotional reciprocity and interpersonal engagement (24, 25) and (b) infant speech perception by interfering in the way basic features of infant-directed speech are expressed and transferred to infants and by obscuring, or reducing infant perception of intersensory coherence, speech intelligibility and maintainance of infant attention [(26), as cited in (27, 28)].

On this ground, as the pandemic continues, the adverse impact on maternal mental health may has long-term effects and powerful influence not only for the generation of infants born during the pandemic, but also for future generations to come (5). We are seriously concerned on this issue because the results of a limited number of the first relevant studies confirm the impact of negatively affected perinatal maternal stress on infant development in the pandemic condition (2, 29–32).

**Figure 1:**
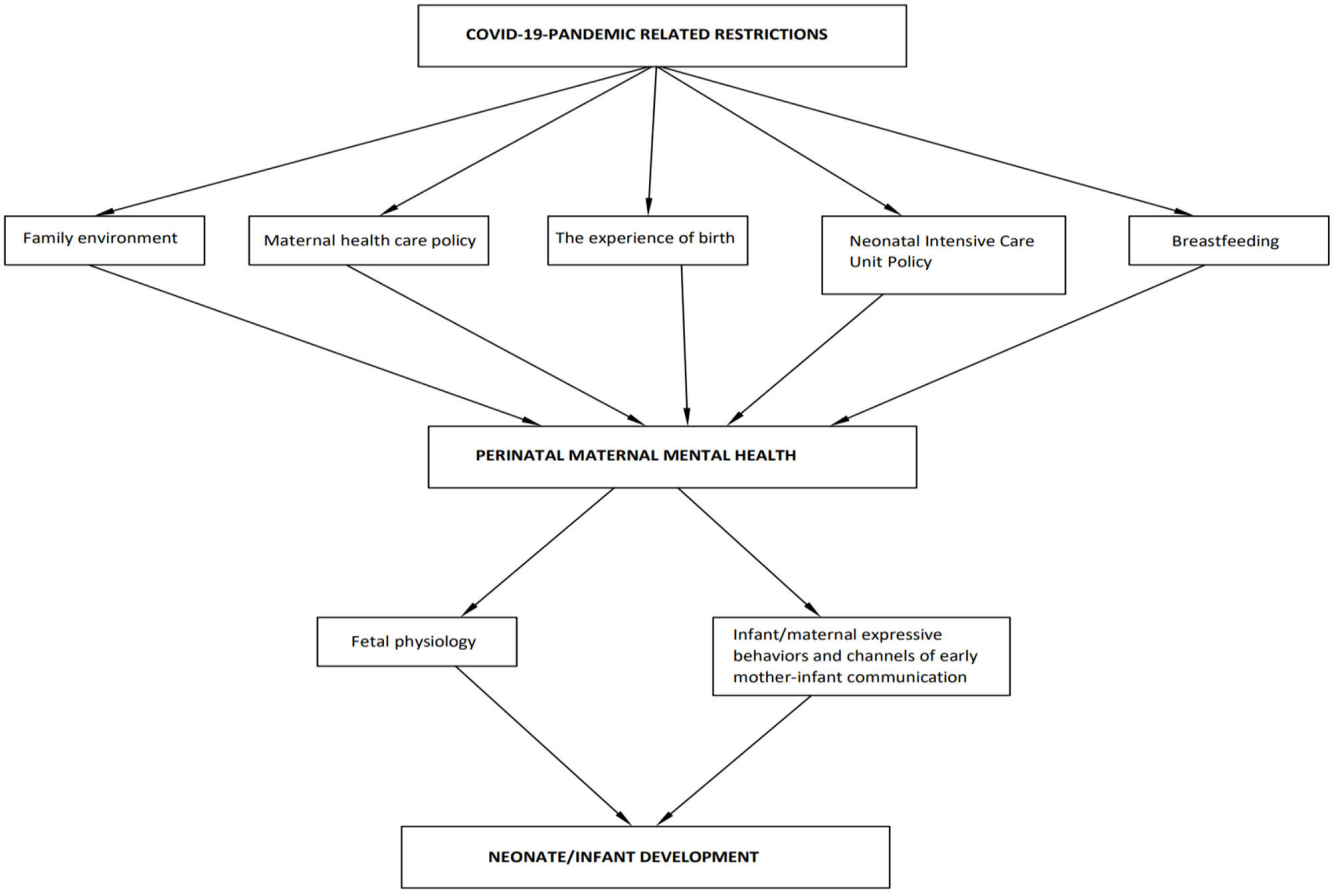
Factors that may affect perinatal maternal mental health through pandemic-related restrictions and implications for neonate and infant development.

The WHO considers maternal mental health as a global health priority (33). Despite that, in this unprecedented time, mental health issues may have been overshadowed by more pressing issues in health care (34). What is more, relevant literature has mainly focused on the impact as an outcome of the pandemic on postpartum maternal emotional wellbeing. A growing body of research investigates women's mental health also during pregnancy (35). In the meanwhile, the factors that may affect perinatal maternal mental health and their connection with infant development have been discussed only in fragments. From an holistic perspective, this review comes to fill in this gap in the literature by discussing the possible factors that may affect perinatal maternal mental health through pandemic-related restrictions, and by highlighting the adverse implications of negatively affected maternal emotional wellbeing on infant development of the COVID-19 generation (see Figure 1).

### Search Strategy

A strategy was formulated and literature searches were conducted from March 2020 to March 2022. The following electronic databases were searched: Web of Science, APA PsycINFO, Academic Search Ultimate, and JSTOR. Search was restricted to papers published in English language, with no restriction on the year of publication. Editorials, opinion papers, empirical studies, reviews, systematic reviews, and meta-analyses were eligible. In this review, we excluded publications which investigated: (a) the way the pandemic may has affected maternal mental health of preschoolers, school-aged children and adolescents and (b) research with a focus on maternal mental health of children, or adolescents with atypical development. Thus, we included only articles with a focus on the way the pandemic has affected perinatal maternal mental health of typically developing neonates and infants. For the first five subsections of the first objective of this review, literature search included the combined use of the keywords: “COVID-19 or coronavirus or 2019-ncov or sars-cov-2 or cov-19” with each of the following terms: “maternal mental health or postnatal mental health or perinatal mental health,” “family environment family relationships or family dynamics or family functioning”/“family cohesion,” “maternal care or maternal health or reproductive health,” “maternal health or pregnancy or perinatal,” “breastfeeding or breast-feeding or infant feeding or lactation or lactating,” “birth experience or womens' feelings about birth or birth satisfaction,” and “neonatal intensive care unit or nicu or baby unit or newborn intensive care.” For the sixth subsection of the first objective of this review, literature search included the combined use of “COVID-19 or coronavirus or 2019-ncov or sars-cov-2 or cov-19” with “maternal mental health or postnatal mental health or perinatal mental health” and “infant development.” Thus, for the coherence of this review, we restricted the presentation of these studies only to those that provided evidence on infant developmental outcomes in relation to perinatal maternal mental health in the first months of life. For the second objective of this review, search terms included the combined use of “maternal depression or postpartum depression or perinatal depression” or “maternal anxiety or postnatal anxiety or perinatal anxiety” or “maternal stress during pregnancy” with each of the following terms: “infant development,” “fetal systems,” “fetal physiology,” “mother-infant interaction or early interaction,” “mother-infant attachment,” “maternal sensitivity or parental sensitivity or caregiver sensitivity.”

## Possible Factors That may Have Affected Negatively Perinatal Mental Health Through the Pandemic-Related Restrictions

### Family Environment and Parental Mental Health

The lockdowns and shutdown policies related to COVID-19 have led to economic difficulties, instability or loss of job and uncertainty for the future economic status. The introduction of strict measures changed considerably the daily routine of citizen. Further, COVID-19 has been posing a threat to interpersonal interactions and relationships due to both a limited physical proximity with one's not cohabiting family members/friends, and at the same time, a forced and prolonged cohabitation with members of one's family (1, 36). Family environment/function has a significant impact on the mental health of its members. Under a crisis condition, the family environment may affect the interaction of negative emotion among its members (37). The unusual family environment has been associated with mental disorders among pregnant women. The mother's perinatal mental health is influenced by her family environment and compatibility between them is very important (38). Parental depression can be passed on to children through poor family function (39).

In connection to these, findings coming from limited studies in different regions provide evidence of a negative impact of the COVID-19 pandemic on the *family environment* and *parental (maternal and paternal) mental health* (1, 38, 40–46).

In particular, in China, Xie et al. (38) showed that, compared to the pre-pandemic period, the scores of cohesion^2^ and independence in families were much lower during the pandemic. The scores for family cohesion were negatively related with depression, anxiety, and hostility symptoms. Similarly, Li et al. (45) confirmed negative correlations between depressive symptoms and family cohesion/adaptability. In Singapore, parents with greater COVID-19-related concerns reported higher stress and less closeness to their children (41). In USA, Feinberg et al. (42) reported deteriorations in family wellbeing and parent depression in the first months of the pandemic, compared to the period preceding it, and a moderate decline in co-parenting quality. Bate et al. (40) confirmed increased levels of parents' self-reported depression and anxiety symptoms during the pandemic. Higher conflict in the parent-child relationship strengthened the positive links between parent and child emotional health issues. In Canada, since the onset of the COVID-19, compared to respondents without children (35.6%), a significantly higher proportion of parents reported worse mental health (44.3%) with high levels of anxiety, worry, stress and boredom. 28.3% of parents reported being stressed while facing challenges in the relationship with their partner. Regarding sources of support, 47.6% of parents reported connecting with those in the household (43). In the same cultural context, COVID-19 health anxiety was found to impair family engagement because of the increased emotion suppression and the lack of psychological need fulfillment (47). In Spain (one of the worst affected European countries), Gunther-Bel's et al. qualitative analysis (44) confirmed elevated levels of state anxiety during lockdown and provide mixed results on perceived changes in family dynamics. In particular, prevalence of improvement themes (61.7%) outnumbered deterioration themes (41.0%). For parents with children at home, *family (re)connection* was cited most often among the improvement themes while *unbalanced needs* were most frequent among deterioration themes. Despite the fact that marital functioning for couples with children systematically improved with days in lockdown, this was not the case for parenting functions. In Australia, Westrupp et al. (46) showed that, compared to estimates in the pre-pandemic period, during the COVID-19 parents reported higher levels of depression, anxiety and stress, higher parenting irritability and lower levels of family positive expressiveness. In Italy, Donato et al. (1) confirmed that COVID-19 concerns threaten individuals' psychological wellbeing and showed that explicit stress communication^3^ in the couple and responses in dyadic coping mediated the link between COVID-19-related concerns and parents' mental health.

### Health Maternity Care Policy

Basic health services worldwide have been heterogeneously affected by the COVID-19 pandemic. There is a variation in strategies adopted to maintain continuity of maternal health services (49). There is a complex organizational response to COVID-19 in maternal services. In some cases these responses are in direct contraversion of COVID-19 recommendations from relevant organizations. These practices may affect negatively, both physically and psychologically, mothers along with their infants as well as medical staff caring for childbearing women and their families (50–53).

In particular, as a response to COVID-19 crisis, limits have been placed on antenatal classes' attendance by pregnant women, decrease of presentations of obstetric-related conditions to the emergency departments, restrictions on health care tests and treatments availability, in both ante- and postnatal care while companionship for birth and postnatal visiting have been prohibited. Giving birth in hospitals full of SARS-CoV-2 infected patients increase further maternal worries for the possibility of their and their infants' infection with adverse physical and psychological implications for both of them. Expectant mothers' reluctance to attend and delay in seeking treatment, due to the fear of being exposed to the virus, may result in poorer outcomes. Moreover, in cases of maternal SARS-CoV-2 infection, forced separation of mothers and infants for up to 14 days has been reported. This prohibited immediate and uninterrupted skin-to-skin contact. Further, a lack of opportunity to support mothers to initiate breastfeeding in the first hour after birth along with non-acceptance of breastmilk donation have been evidenced. These restrictions have a negative effect on mother's mood, self-esteem, self-confidence and confidence in their abilities related to their infant's care. Standard precautions (such as hand hygiene, use of medical mask, routine disinfection) applied by mothers with suspected, or confirmed COVID-19, who must take care of their infants by themselves, may impose psychological demands on new mothers and may complicate the early mother-infant relationship (51–56).

### The Experience of Birth

During the pandemic, and especially during the lockdown period, limited access to formal, and informal support network along with medical conditions and risk factors may have shaped adversely the experience of birth (35).

In connection to these, limitations set by the pandemic may cause a shift in the way mothers and fathers experience birth. Thus, birth experience may shift from being a “couple event”—based on “togetherness”—to being in “singleness,” placing a barrier within the couple and within the newly born family. Father's stress combined with that of mother in the perinatal period may has implications for infant development (36).

Pregnant mental health is at the core of the following interrelated factors that have been identified as affecting the subjective childbirth experience: pregnant psychological wellbeing, personal history of maternal mental illness, pregnancy complications, fear of childbirth, support and relationship with the partner, fear of health, the first moments with the baby/mother-infant bond (skin-to-skin contact, breastfeeding), previous birth experiences, perceived control, birth plan compliance and medical-obstetric dimensions (33, 35, 57, 58).

In particular, COVID-19 pandemic has *affected adversely all above interconnected factors* that contribute to the subjective childbirth experience (33, 35, 57, 58). Women who gave birth, or pregnant women during the current pandemic, are at greater risk of reporting *general stress*, isolation and frustration at all phases of pregnancy, birthing and infant care. They are also at greater risk of manifesting *depressive, anxiety, or post-traumatic symptoms* (59, 60). In the pandemic condition, depression and anxiety have been negatively related to birth satisfaction (61). New-mothers with *earlier psychological disorders* and *complications during pregnancy* were more likely to suffer from trait anxiety and postpartum depression, to develop a postpartum post-traumatic stress disorder and to have perceived childbirth as a negative experience (35). In the period preceding the pandemic, *the fear of childbirth* was associated with anticipation, impatience, joy and encounter. However, during the pandemic fear was correlated with sadness, loneliness, inability, sense of isolation and constriction (58). For more than half of expectant mothers, fear of childbirth is above the cutoff value while 32% of women reported a negative childbirth experience (35). *Lack of support* from the partner has arisen as a major issue affecting adversely women's pregnancy and childbirth experience under the restrictions imposed due to the pandemic. Pregnant women were more likely to suffer from state anxiety and to have intense fear of childbirth if they believed that their partner could not be present at childbirth, or had not been present during delivery (35). During the earliest months of the pandemic, higher birth satisfaction has been associated with having a birth partner present (61). COVID-19 has intensified *the protective response of women for those around them* (13, 16, 62). This shift of focus to others' health may increase the risk of maternal mental health problems (13). Regarding the first moments with their infants, Del Rio et al. (50) showed that 43.5% of infants did not receive maternal *skin-to-skin contact after birth*. After the quarantine termination, 49.1% of SARS-CoV-2 infected mothers chose to prolong mother-baby separation. In the first months of the pandemic, separation from the infant has been negatively associated with birth satisfaction (61). The combined impact of isolation with the feeling of the newborn as “fragile” caused tension and mistrust (59). Ravaldi et al. (58) showed that before the pandemic, *previous birth experiences* were associated with positive expectations for birth but during the pandemic the same experiences changed to feelings of danger, anxiety and loneliness. Pandemic situation has been associated with *birth plan* changes (e.g., place of birth, presence of birth partner) (63) which may negatively affect their birth experience and the sense of personal achievement and control (33). Regarding *medical-obstetric dimensions*, pandemic-related health care policy and maternity care practices have a negative impact on birthing women's perceptions of safety and support (64). Women who gave birth during the pandemic gave a worse rating of the quality of care they received (65).

### Neonatal Intensive Care Unit Policy

The pandemic-related restrictions in NICU vary widely depending on local infection rates, availability of personal protective equipment and the structure and layout of the NICU (66). In the meanwhile, restrictions in NICU parental presence have been widely adopted (67). COVID-19-related policies that impose restrictions on components of parent presence in NICU (e.g., who can be present, how many people can be present, when they can be present) may inhibit the concept of parents as “partners in care” against the Family Centered Care (FCC)/Family Integrated Care (FIC) model concepts (66, 68–70) with adverse effects on the components of FCC, on infants, new parents and stress-related consequences in health professionals (68).

FCC/FIC—the gold standard in healthcare—has been incorporated into neonatal intensive care units. This kind of care encourages and empowers parents to play an active role in the caregiving of their child, while cooperating with staff and taking part in the decision making for their infants. Providing parents with the opportunity to exercise their role of primary caregivers brings benefits in their emotional health with short- and long-term positive implications for infant development such as: improved weight gain, increase in the incidence of breastfeeding, decreased parental stress and increased parental satisfaction rates, improved neurodevelopmental outcomes (through parent-infant skin-to-skin contact) and the development of parent-infant bonding. Further, promoting parental mental health will also support health professional's wellbeing. In this context, FCC requires that parents are not labeled as “visitors” but rather as “partners in care,” or they provide most of the care for their infant (66, 68–70).

Limiting parent presence may contribute in additionally aggravating the psychological distress of NICU family, a vulnerable population due to trauma of separation from the infant along with stress for their medical condition (54, 67, 71). The experience of parenthood in NICU has been negatively affected by the restrictions in parental presence time and physical contact of parents with their infant and additional concerns for their child's health. Parent presence due to isolation recommendations are connected with restrictions on parent-newborn contact, loss of opportunities for interaction and for provision of parental care. Restrictions may impact adversely breastfeeding and may cause additional emotional disturbance on parental pandemic-related preexisting heightened anxiety with adverse effects on parent-infant bonding and infant neurodevelopment. What is more, the restriction to fathers' access to the NICU acted as a significant obstacle to early infant-father bonding and led to loneliness and isolation by the mothers (66, 68, 71–73).

Travel restrictions due to the pandemic constituted an obstacle for family members' presence to provide postpartum social support. Thus, many women are left feeling isolated and alone, a condition that potentially contributes to risk of developing perinatal anxiety and mood disorders (74). Less support from family and friends and loneliness have been frequently reported by parents in NICU (71).

The majority of the newborns born by SARS-CoV-2 infected mothers were followed in isolation rooms in the NICU, others were monitored with a distance of 2 m away from the mother, or cared by family members in a separate room (Yekta 72). Policies that impose limitations in early neonate-mother tactile interaction may disrupt the previously established fetus-mother communication and may have detrimental effects on all domains of infant development, parental mental health and on the quality of spouses' relationship (75–83).

Early skin-to-skin care has been related to positive influences on maternal mental health and sensitivity, oxytocin levels (which facilitates mother-infant bonding with long-term effects), lower pain perception, improved self-efficacy of mothers of premature infants, growth of parental self-esteem, and higher maternal satisfaction (77, 84–86). Early skin-to-skin care has also beneficial effects for father-infant attachment scores/interactive experiences, for fathers themselves at a biochemical level, and on their mental health which can have an indirect positive impact on maternal domains (exclusive breastfeeding and care-giving-related hormones), and increases spouses' mutual understanding (87–94).

In addition, NICU staff members experience a sudden and continuous environmental stressor since they are further affected by a number of factors that seem to increase even more their psychological stress such as: moral distress when limitations beyond their control make them unable to take decisions according to their own values, the values of the patient's family, or the values of FCC; and difficulties in finding a balance between meeting the emotional needs of hospitalized infants and their families while also safeguarding their own health (52, 54, 68).

Further, it has to be emphasized that personal protection equipment wearing, virtual consultations and online antenatal education cause disruption to interpersonal communication and limit supportive touch between NICU staff and parents (52). Face-to-face psychological support of pregnant and new mothers by mental health professional is equally important as physical checks. A trusting relationship between professionals and families is a prerequisite for good quality maternal and family care. Dynamics of interpersonal communication, such as good eye contact, touch, and tone, are essential elements of care (52). Under conditions of substantial mental health burden of both healthcare workers and new parents, limitation of interpersonal engagement may aggravate even more the risk of emotional exhaustion of pregnant women/new mothers and their families.

### Breastfeeding

The nutritional and physical health benefits of breastfeeding for infants and children are well-established. Accumulating research shows the long-term effects of breastfeeding on brain, cognitive and socio-emotional development of children and on mental health of mothers (95).

In the course of COVID-19 pandemic, the main scientific and public institutions (e.g., WHO, UNICEF) advice to facilitate mother-infant interactions and to support breastfeeding initiation even in cases in which a mothers has been virus infected as long as clinical conditions permit it (63, 96, 97). In the meanwhile, other institutions highlight the risk of virus transmission and recommend maternal separation precluding breastfeeding (98). So far, though emerging evidence suggests that vertical transmission is possible, there is not enough scientific evidence to unequivocally state the possibility of SARS-CoV-2 mother-infant transmission via breastmilk. However, infection transmission risk is attributed to close contact between neonate and mother with suspected, or confirmed infection during breastfeeding [see (99–101) for reviews].

Maternal mental health constitutes a core factor related to face-to-face breastfeeding support, skin-to-skin contact after birth and partner's support, all interrelated factors connected to breastfeeding, one aspect of the gold standard infant care (102–104). There is evidence that most of these factors have been affected adversely by the COVID-19 pandemic.

Regarding *maternal mental health*, under the pandemic condition, a limited number of studies shows that breastfeeding mothers reported that they experienced anxiety, depression, isolation, loneliness and distress for not being able to see their family during the lockdown (63, 105–107). Worries about the safety of breastfeeding were commonly mentioned but, at the same time, exclusive breastfeeding was a protective factor to maternal mental health (102, 106). Continued breastfeeding support is a key for breastfeeding success while the quality *of breastfeeding support* is important for both breastfeeding promotion and maternal mental health (63, 103, 108). The *lack of face-to-face health services* and lack/decrease of support for breastfeeding has been mentioned as one of the main concerns of breastfeeding mothers, the most common reason for breastfeeding cessation, a frequent maternal response related to feeding plans changes and as a factor that negatively impacted breastfeeding experience (59, 63, 102, 105). However, the absence of recommendations on breastfeeding support and lack of support has led to reduced compliance to the recommendations of main scientific institutions suggesting breastfeeding initiation. This may has affected adversely both maternal mental health and the rate of breastfeeding (50, 51, 109). However, mothers experience breastfeeding heterogeneously since 41.8% felt that breastfeeding was protected due to the lockdown but 27% of mothers reported a negative impact of lockdown upon their breastfeeding experience. An intense focus on feeding which made them feeling overwhelmed by the breastfeeding experience and a *lack of face-to-face support* were some of the barriers placed in their way (102). As for *partner support*, both women who delivered before and during the lockdown reported that the main source of infant feeding support is the partner (63). Mothers who reported a positive impact of the pandemic on breastfeeding mentioned greater partner support. Shared care was felt to strengthen the new parent relationship and to increase bonds between partner and baby. Mothers who reported an adverse effect of the pandemic on breastfeeding talked about the isolation they felt which had a negative impact on their wellbeing and mental health (102). Regarding the connection of skin-to-skin contact and breastfeeding, in the course of COVID-19 pandemic, there is evidence for a strong negative correlation between exclusive breastfeeding at discharge and mother-newborn separation at birth (50). The implications of early mother-neonate separation have been discussed above.

### COVID-19 Pandemic, Maternal Mental Health, and Infant Development: First Results

To our knowledge, the results of a limited number of relevant studies from different regions confirm our concerns on the negative effect of adversely affected perinatal maternal stress on infant development in the pandemic condition.

In Italy, the first longitudinal study (32) that documented the short-term implications of COVID-19 pandemic-related stress on infant's temperament at 3 months, showed that infant's regulatory capacity was linked with less parenting stress and more mother-infant bonding. In Japan, mother-infant bonding was worse one month after birth among mothers who gave birth during the COVID-19 pandemic compared to those who gave birth in the same period of the previous year (110). Maternal mental health problems have been related to long-term risks for the establishment of mother-infant bonding (30). In Portugal, compared to mothers who gave birth before the pandemic, mothers of 0–12 month-old infants who gave birth during the COVID-19 pandemic presented lower levels of emotional awareness of the child and a more impaired mother-infant bonding. A more impaired mother-infant bonding was associated with higher levels of parenting stress and lower levels of mindful parenting dimensions. Maternal mental health problems may prevent maternal adoption of a mindful parenting practice (30).

An online survey, mainly in European countries, showed an acute decrease in sleep quality (which plays a crucial role in brain maturation) in 0–35-month-old infants and 36–71-month preschool children in April 2020. At two-follow up assessments (May/June 2020), this effect largely disappeared. Caregiver's stress due to the confinement was identified as the dominant factor with a negative impact on children's sleep. In the meanwhile, protective factors influencing children's sleep quality included caregiver's mindful techniques, childcare and the presence of siblings/pets (111). What is more, children (aged 0–4) with parents scoring higher on separation anxiety showed more distress after child care center reopening. There was a positive correlation between concurrent child and parental distress after reopening (29).

In Serbia, Jelicic's et al. follow up study (31) showed medium and high levels of maternal anxiety among 142 third-semester pregnant women during the COVID-19 pandemic, and a high level of perceived social support. The study showed a positive correlation between maternal trait anxiety and child's socio-emotional status at 12 months.

Lastly, there is evidence that COVID-19-related prenatal stress was significantly correlated with higher infants' SLC6A4 methylation (which occurs at the level of stress-related genomic portions). SLC6A4 methylation was negatively associated with the infants' positive affect at 3 months (2).

## Possible Effects of Maternal Depression and Anxiety During the COVID-19 Pandemic on Fetal and Infant Development

There is evidence that maternal stress impacts *fetal central and autonomic nervous system function* (ANS) (15) and both *maternal hypothalamic-pituitary-adrenocortical axis (HPA) activity and fetal HPA development* (19, 20). The early structures of *the developing limbic system (e.g., amygdala and hippocampus)* may also be influenced by the maternal stress (21). What is more, heightened CpG-specific SLC6A4 methylation has been evidenced for infants exposed to prenatal maternal depression and stress. This is important given that heightened SLC6A4 methylation constitutes a potential biomarker of early adverse experiences (2). Prenatal stress exposure has also been related to *physical health-related outcomes* [(14, 18, 112–114), as cited in (20, 115, 116)].

Taken the above evidence together, it is critical that the development of fetal systems [ANS, HPA development and brain structures of the limbic system (amygdala and the hippocampus)] that have been reported to be affected by maternal prenatal stress are also potentially related factors and causes of neuropsychiatric disorders [depression, anxiety, behavioral dysfunction, attention-deficit hyperactivity disorder (ADHD), autism spectrum disorder] in children (21). Though most children are not affected by prenatal stress, partly due to differential genetic susceptibilities (117), we express our concern that the COVID-19 pandemic may contribute into a further increase in the incidence of neuropsychiatric disorders in children, and later in life, as this may be reported in the coming years.

What is more, research provides evidence that maternal depression negatively affects both infant and maternal expressive behaviors. Maternal depression disrupts all channels of early infant-mother communication and parameters of fine-grained interactive temporal coordination along with the dyad's capacity to mutually regulate the interaction (118–125). What is more, maternal depression has a negative effect on mother-infant affective and behavioral synchrony, bonding, attachment, mutual attunement and negatively affects positive enrichment activities and care for the infant, infant health and sleep, breastfeeding and its parameters (e.g., duration, timing exclusivity, satisfaction, confidence and weaning) [(22, 23, 123) for recent reviews]. On this ground, maternal depression has been linked with delays and poor infant motor, social, cognitive and language development and difficulties in emotional self-regulation. Maternal depression has been associated with short and long-term adverse consequences for mothers' physical and psychological health, partner relationships, sexuality and social relationships [see (22, 23) for recent reviews].

Recent evidence on the impact of maternal anxiety on mother-infant interactions shows that high levels of maternal state anxiety significantly predicted a lower score on the sensitivity scale (126), reduced emotional tone and increased level of non-contingent maternal comments during interaction to their infants (127). Anxious mothers present greater intrusiveness (128) and they do not adapt to the infant's moment-by-moment signals (129). Infants of anxious mothers seem less communicative, less emotional during social challenges (130) and they score less optimally on social engagement (129). Maternal anxiety has been positively associated with infant negativity and with mismatches in which infant was in positive affect and mother was in negative affect, or infant expressed negative emotion and mother was in a neutral state (131).

What is more, face masks may aggravate even more the already disrupted channels of early infant-mother face-to-face interaction. In connection to this, in relation to non-depressed mothers, depressed mothers manifest more flat and negative affect, less positive affect and increased gaze focus at the infants (132). Infants of depressed and anxious mothers are likely to encounter even fewer opportunities to observe and imitate facial expressions of emotion (133). Infants of depressed mothers need more trials and take almost twice as long to habituate to their mother's face and voice compared to infants of non-depressed mothers (134).

## Discussion

We reviewed possible factors that may have affected negatively perinatal mental health through the pandemic-related restrictions. We presented the implications of adversely affected maternal emotional wellbeing on infant development.

On the basis of the above review, we would like to note the following:

1. It is our obligation to emphasize that evidence-based promotion of new family mental health during the COVID-19 pandemic is needed to be integrated within the health system at a multi-layered level in the prenatal, intrapartum, postnatal period and in infancy/early parenthood (135, 136). Protective factors, including partner/social support (137) must be taken into consideration. All healthcare providers involved with birth and NICU staff must give even more active support to new families (36, 54). What is more, maternal mental health screening during the pandemic condition has been highlighted as an essential issue (137). It is important for nurses in obstetric units to identify stressors of pregnant women in the course of prenatal care and provide resources to manage/reduce their impact. Providers in outpatient clinics should consider synchronous group prenatal telehealth care visits that may provide support for pregnant women by creating a sense of community (74).
2. High priority should be given to the preservation of family-centered care principles with emphasis on parents' presence in the NICU, parent-infant physical and emotional closeness and parental involvement in the infant's care (54). Toward this direction, while strict restrictions on parental presence were initially adopted to prevent infection spread in the NICU, recently there is a relaxation of such restrictions in favor of parent-infant contact (71). It is the responsibility of hospital systems to ensure that family-infant communication can continue to be supported in the safest manner possible (138). Current recommendations are being modified on a case-by case basis. For mothers in good clinical condition, the separation of the mother-child pair might be not recommended. The recommendations for infected neonates vary from isolated admission without caregivers to strategies adapted to the clinical situation of the infant, but with parental accompaniment (68). When parents cannot be in the NICU, it is crucial that they must be supported to see their baby via video (54). Video-technology interventions showed parental appreciation of being able to see their infant when they could not be in the NICU. Parental ability to visualize their infant reduced stress and anxiety. Videoconferencing seems to be helpful and meaningful to parents [see (72) for a full discussion]. Further, NICU systems should implement evidence-based assessment and treatment for parental distress while providing peer support for parents and voice calls [(54); see in (72) for a discussion on the benefits and the drawbacks of online support groups].
3. We are seriously concerned that the COVID-19 healthcare emergency may be “…a hidden pandemic of developmental psychopathology” [(32), p. 7]. There is an urgent need for more investment to research with the aim to evaluate the way the pandemic is affecting maternal mental health and on the impact of poor maternal mental health on young infants' developing brains. Also, there is a need to follow up these children and their families in order to mitigate the COVID-19 pandemic effects in the long-term. Health authorities and government have to treat this as a public health issue, and not as a condition with short-term effect (34, 139).

## Conclusion

We highlighted the possible factors that may have affected negatively perinatal mental health through the pandemic-related restrictions. We presented the implications of adversely affected maternal emotional wellbeing on infant development. It is critical to extend with more research our understanding of the way the pandemic is affecting maternal mental health and the impact of poor maternal mental health on infant development. The implications of the adverse maternal wellbeing on infant development under the pandemic condition call for nationwide policies and evidence-based interventions. These interventions have to be integrated within the health system for prenatal and postpartum care in an effort to promote new family wellbeing and infant development. Interventions for improving perinatal maternal mental health is needed to be adapted in the “new normal” of the current situation. Maternal wellbeing and the implications of it on infant development should be priority areas to be included in COVID-19 related policy guidelines.

## Author Contributions

TK and EH contributed equally to the writing of this review. All authors contributed to the article and approved the submitted version.

## Conflict of Interest

The authors declare that the research was conducted in the absence of any commercial or financial relationships that could be construed as a potential conflict of interest.

## Publisher's Note

All claims expressed in this article are solely those of the authors and do not necessarily represent those of their affiliated organizations, or those of the publisher, the editors and the reviewers. Any product that may be evaluated in this article, or claim that may be made by its manufacturer, is not guaranteed or endorsed by the publisher.

## Footnotes

^1^These studies compared mental health between pregnant and non-pregnant women, or mental health of pregnant women during the pandemic with pre-pandemic period, or they provided quantitative evidence of anxiety and depression symptomatology of pregnant women in different countries.

^2^The Olson Circumplex Model explains family function and the way family systems will change in response to a crisis. It includes two dimensions: family cohesion and family adaptability. “Family cohesion is defined as the emotional bond that family members have toward one another” [(37), p.145]. “Family adaptability is the amount of change in its leadership, role relationships and relationship rules” [(37), p. 147]. The four levels of cohesion range from disengaged (very low) to separated (low to moderate) to connected (moderate to high) to enmeshed (very high). The four levels of adaptability range from rigid (very low) to structured (low to moderate) to flexible (moderate to high) to chaotic (very high). The balanced levels of cohesion (separated and connected) and adaptability (structured and flexible) are considered as optimal family functioning while the unbalanced (disengaged or enmeshed/ rigid or chaotic) are seen as problematic for relationships in the long-term. Balanced types of couples and families will have more positive communication compared to unbalanced systems) (37).

^3^According to Bodenmann's Systemic-Transactional Model of dyadic coping (48), dyadic stress is observed when partners are affected by a stressor and the source of stress is defined as common. In order to cope against dyadic stress, partners initiate a dyadic coping process. A dyadic coping process is the interplay between both partners' stress, their coping reactions along with proper common responses to the dyadic stressor. Stress communication is constitutes the first step in the dyadic coping process (1).
